# Health outcomes and female genital mutilation/cutting: how much is due to the cutting itself?

**DOI:** 10.1038/s41443-022-00661-6

**Published:** 2023-01-04

**Authors:** Crista E. Johnson-Agbakwu, Georgia J. Michlig, Sophia Koukoui, Adeyinka M. Akinsulure-Smith, Danielle S. Jacobson

**Affiliations:** 1 Southwest Interdisciplinary Research Center, Watts College of Public Service and Community Solutions, Arizona State University, Phoenix, AZ USA; 2 Refugee Women’s Health Clinic, Valleywise Health, Phoenix, AZ USA; 3 Creighton University School of Medicine, Phoenix Regional Campus, Phoenix, AZ USA; 4 District Medical Group, Phoenix, AZ USA; 5 Johns Hopkins Bloomberg School of Public Health, Baltimore, MD USA; 6 Université de Montréal, Psychology Department, Montreal, QC Canada; 7 CIUSS Centre Ouest-de-l’ile-de-Montréal-Sherpa, Montreal, QC Canada; 8 The City College of New York, Department of Psychology, New York, NY USA; 9 City University of New York, The Graduate Center, New York, NY USA; 10 Dalla Lana School of Public Health, University of Toronto, Toronto, ON Canada

**Keywords:** Human behaviour, Quality of life

## Abstract

While Female Genital Mutilation/Cutting (FGM/C) continues to garner global attention, FGM/C-affected migrant communities, who are often racialized minorities in the U.S., face additional challenges which may impact their physical and mental health and well-being. It has been proposed that an overly narrow focus on the female genitalia or FGM/C status alone, while ignoring the wider social experiences and perceptions of affected migrant women, will result in incomplete or misleading conclusions about the relationship between FGM/C and migrant women’s health. A cross-sectional study was conducted across two waves of Somali and Somali Bantu women living in the United States, (*n* = 879 [wave 1], *n* = 654 [wave 2]). Socio-demographics, self-reported FGM/C status, perceived psychological distress, and self-reported FGM/C-related health morbidity was examined against self-reported experiences of everyday discrimination and perceived psychosocial support. In statistical models including age and educational attainment as potentially confounding socio-demographic variables, as well as self-reported FGM/C status, self-reported discrimination, and perceived psychosocial support, self-reported discrimination was the variable most strongly associated with poor physical health and psychological distress (i.e., FGM/C-related health morbidity and psychological distress), with greater perceived psychosocial support negatively associated with psychological distress, when controlling for all the other variables in the model. FGM/C status was not significantly associated with either outcome. Discrimination, more frequently reported among ‘No FGM/C’ (i.e., genitally intact or unmodified) women, was most frequently perceived as linked to religion and ethnicity. Our findings are consistent with views that discrimination drives negative outcomes. In this population, discrimination may include the *‘quadruple jeopardy’* of intersecting relationships among gender, race, religion, and migration status. We find that self-reported experiences of discrimination—and not FGM/C status per se—is associated with adverse physical and mental health consequences in our sample drawn from Somali migrant communities living in the United States, and that social support may help to mitigate these consequences. Our findings thus reinforce calls to better contextualize the relationship between FGM/C and measures of health and well-being among Somali women in the United States (regardless of their FGM/C status), taking psychosocial factors more centrally into account.

Clinical Trials.Gov ID no. NCT03249649, Study ID no. 5252. Public website: https://clinicaltrials.gov/ct2/show/NCT03249649

## Introduction

Female Genital Mutilation/Cutting (FGM/C) increasingly garners global attention due to trends in migration patterns from high to low prevalence regions. It is estimated that over 200 million women and girls have experienced medically unnecessary genital cutting of some kind, with approximately 4 million girls at risk of such cutting annually [1]. FGM/C carries numerous short- and long-term health risks, ranging from mild to severe; specific risks depend on the type and extent of cutting, the degree to which the practice has been medicalized, and other factors. Adverse outcomes that have been associated with FGM/C of various types include obstetric, gynecologic, sexual, and psychologic harms to health and well-being [2]. Among those who endorse FGM/C, it is widely believed that the practice helps to imbue a sense of belonging, often related to cultural identity (e.g., ethnic, religious, or gender-based), while also increasing one’s social status, respectability, and, among other things, perceived sexual virtue or desirability according to local standards (e.g., enhancing sexual power or agency in some groups; tempering “excess” sexual desire or promoting virginity in others) [3–6]. In any case, where FGM/C is a dominant social norm or seen as a prerequisite for important life goods (e.g., recognition as an adult, eligibility for marriage), a lack of FGM/C is often heavily stigmatized, with negative social consequences for challenging the practice or associated cultural scripts [7–9].

Most of the published literature on FGM/C has been produced from within a Western colonialist framework, typically drawing on assumptions about gender, health, identity, and sexuality that may not translate to every cultural context [5, 10] 1. Postcolonial African feminist discourse enables a centering of the lived experiences of women affected by FGM/C across intersectional identities along axes of power, privilege and/or oppression and across socio-historical divides [15]. We draw upon the latter discourse to frame our discussion in this paper.

The notion of intersectionality, which encompasses the concept of multiple, interlocking systems of political identities and racial, sexual, and patriarchal oppression was first introduced by the Combahee River Collective, a Black lesbian feminist socialist organization that was active from 1974 – 1980 [16]. From 1981, the overlapping influence of race, class, and gender in perpetuating oppression began to be popularized in the early writings of the late Black feminist writer and activist bell hooks [17]. It was not until 1989 when the term, intersectionality, became formally codified as an analytical framework by Kimberlé Crenshaw [18]. Intersectionality posits that interconnected aspects of a person’s social and political identities coalesces to create distinct patterns of susceptibility to discrimination, oppression, and privilege across multiple modes of advantage and disadvantage. Within the context of FGM/C, it is important to note that the practice extends well beyond the African sub-continent, being highly prevalent throughout regions of the Middle East and South and South-East Asia [19]. Nevertheless, when considering the historic and socio-political context of racialized communities of color [20], especially in the United States, the state of African migrants must be given due attention given the particular manner in which they have been represented within Western culture.

We do not suggest that there is anything monolithic about these groups, or that they have followed the very same migratory pathways. Rather, each community member occupies a unique space at the intersection of race, gender, religion, age, sexuality, and geography. At the same time, women in these diverse communities are broadly subjected to, and affected by, the socio-medical *‘othering’* of Black women’s bodies [21]. This can involve, among other things, treating their bodies as exotic curiosities or even objects of medical voyeurism, perhaps especially when their bodies have been shaped by the culturally unfamiliar practice of FGM/C.

Thus, by ignoring the intersectionality of women affected by FGM/C—for example, by focusing too narrowly on their altered genitalia without adequately considering variability in their wider psychosocial experiences and circumstances —there is risk of reinforcing neocolonial assumptions of cultural hegemony among racialized minorities across the African diaspora [15]. For example, recent scholarly discourse has called into question the World Health Organization’s definition of FGM/C, which effectively “lumps together”—and disparages as mutilating—all medically unnecessary procedures involving “partial or total removal of external female genitalia or other injury to female genital organs” [22] that are associated primarily with “African” cultures, irrespective of cutting severity or consent. Meanwhile, the WHO does not condemn medically unnecessary genital cutting practices that are normative within “Western” cultures, including some that arguably fall within their definition of “FGM/C” (such as female genital “cosmetic” surgeries) [23, 24] and others that are performed on a non-voluntary basis on minors without consent [25–27].

In the Global North, migrant communities of color, which includes FGM/C-affected communities^2^, suffer disproportionate setbacks to health and well-being due to the confluence of sociopolitical, socioeconomic, environmental, linguistic, migration-related barriers, and (other) sociocultural factors. These factors are collectively referred to as social determinants of health (SDoH). Structural racism, defined as societal forces which perpetuate mutually reinforcing systems and racially discriminatory beliefs, values, and distribution of resources, undergirds the SDoH [28]. It influences health policies and practices, and negatively affects migrant communities of color, further widening disparities in timely access to care, use of health services, and patients’ experiences with care. These disparities, in turn, further exacerbate their inequitable health outcomes [29]. FGM/C has become a distinguishing cultural marker for a racialized and socially marginalized subgroup at heightened risk of untoward experiences with health care and resultant deleterious health outcomes.

To shed light on the health and well-being of a diasporic Somali community in the Southwestern U.S., we investigated associations between FGM/C and related obstetric, gynecologic, and sexual health outcomes juxtaposed against overarching experiences of discrimination and social support. While prior evidence has shown that FGM/C status is associated with increased health morbidities including sexual dysfunction, obstetric issues, gynecological and mental health sequelae [2, 30], the objective of this analysis was to explore whether wider psychosocial factors may also contribute to or predict health outcomes, beyond the singular focus on FGM/C. Might other variables besides FGM/C be more significant in affecting health outcomes along certain dimension? Might there even be identifiable benefits associated with belonging to a practicing community? Disentangling the multiple, intersectional aspects of SDoH will be necessary for understanding the distinctive impact of FGM/C on the lived experience of women in migrant communities of color in the Global North. We first examine experiences of discrimination and social support in this population and explore whether variations in these experiences exist between women with and without FGM/C. We then explore through multivariable modeling to what extent FGM/C, discrimination, and/or social support are independently associated with poor self-reported mental and physical health outcomes.

## Subjects and methods

This study was part of a larger effort to improve health services to women affected by FGM/C in Arizona [30]. Institutional Review Board (IRB) approval was secured from Arizona State University (STUDY00005252). Community Based Participatory Research (CBPR) principles are critical when engaging with hard-to-reach communities, particularly on sensitive, value-laden, and taboo topics like FGM/C [31, 32]. Equitable inclusiveness of community partners was a foundational framework that guided all research phases, from conception to implementation, interpretation of findings and dissemination. Longstanding deeply rooted and trust-based community partnerships with the Somali community had been cultivated, nurtured, and sustained for nearly 10 years prior to the present CBPR engagement, wherein the community had already been engaged in meaningful community dialogue and educational outreach on FGM/C [30, 33–36]. Furthermore, health services for FGM/C-affected women and girls had been provided by the first author, who also possessed over 20 years of experience working directly with FGM/C-affected communities and who also delivered cultural sensitivity training to health care providers on engaging with and addressing the unique health care needs of FGM/C-affected communities. Consequently, in this local Somali community, a patient-centered medical home at a public safety net health care system had already been well established for nearly 10 years prior to the recruitment of participants and was anchored in the provision of trauma-informed FGM/C-related care, providing a safe space for open, trust-based, and empathic dialogue on women and girls’ health care.

Within this setting, Somali and Somali Bantu women aged 15 and older, residing in the urban regions of Phoenix or Tucson, Arizona, were recruited to participate across two waves of a cross-sectional Community Health Needs Assessment (CHNA) survey. Informed consent was obtained from all participants, and adolescents aged 15–18 required parental permission (i.e., “proxy consent”) and youth assent. Respondent Driven Sampling (RDS) was used during the initial month of data collection only, after which time dynamic geopolitical shifts in U.S. policy towards migrants created fears within the Somali community concerning study participation, necessitating a shift towards snowball sampling to optimize recruitment [30, 33, 37, 38]. Among the women in wave 1 (*n* = 879), 654 women were matched in wave 2. A total of 915 women were approached for study recruitment, of which 872 gave consent and answered at least 1 question on the first wave of the survey, resulting in a response rate of 95.3%. For the second survey wave, which occurred between approximately 18 months to 24 months after the first wave, we were able to follow-up with 654 of the original 872 sample, with a retention rate of 75%. This high recruitment and retention rate during an extraordinarily tumultuous geo-political and social environment embroiled in intensive anti-migrant animus would not have been achievable without the decade-long, deeply rooted, trust-based relationships and safe spaces that had been cultivated, nurtured, and sustained throughout.

### Study design

CHNA surveys were developed with input by an expert review panel alongside community key informants comprising community leaders who were both women and men, elders, and youth, including religious figures. The surveys were translated, back-translated, and made available in English, Somali and Maay Maay languages. Data collection ensued from February to December 2017 (wave 1), and from December 2018 to June 2019 (wave 2). Data was collected using REDCap (Research Electronic Data Capture) [39] and Qualtrics [40]. Multilingual female Somali community mobilizers (CMs) completed comprehensive training and verbally administered surveys using electronic tablets. Each survey, across both waves, took approximately one hour to complete. A $25 USD incentive was given to each participant upon survey completion.

### Measures

All variables in this analysis are from the wave 2 survey apart from self-reported ethnicity, age, and FGM/C status, which were assessed at baseline. Since the specific date of the follow up survey was not collected, age at wave 2 was calculated by adding 1.5 years to the age given at baseline. Self-reported FGM/C status was facilitated by visual imagery on electronic tablets using the World Health Organization’s typology scheme to enhance the accuracy of FGM/C self-report [41].

The Refugee Health Screener 13 (RHS-13) was utilized to measure clinically significant psychological distress. It is a highly sensitive initial assessment tool to identify individuals suffering from mental health concerns. It assesses symptoms of disorders that are common in refugee populations (PTSD: sensitivity 0.81/specificity 0.87, anxiety: 0.94/0.86, and depression: 0.95/0.89) [42]. This scale has previously been validated in refugee populations to be predictive of PTSD, generalized anxiety disorder, and depression [43–45]. The RHS-13 is scored by summing the thirteen 5-point Likert response questions of the scale and determining the screen positives according to a cutoff value of ≥11. Individuals with any missing RHS-13 data (*n* = 62) were excluded from analysis.

The outcome related to physical health was analyzed dichotomously based on whether the participant had experienced any of 28 gynecological, sexual, or obstetric health concerns in the past two years. Examples include difficulty passing urine, recurrent urinary tract or genital infections, pain with intercourse, difficulty getting pregnant, emergency C-section, and postpartum hemorrhage. Since this variable was assessed based on self-report rather than review of medical records, introducing recall bias, it was determined to treat this variable dichotomously to determine simply whether an experience of poor health in these areas had occurred as opposed to a more granular treatment of the variable.

Perceived social support was measured using the Multidimensional Scale of Perceived Social Support [46], which included twelve 5-point Likert questions representing three subscales of support sources including perceived support from [1] a significant other [2], family, and [3] friends. The validity of this scale has been confirmed among other immigrant and refugee populations, including most recently Chin-Burmese refugees [47] and Arab American immigrant women [48]. Internal reliability of the scale demonstrated a strong Cronbach’s alpha of 0.966. All scale items were totaled and averaged to present a mean score. Subscale items were also uniquely totaled and averaged to present subscale mean scores. Participants’ missing data for one or more scale items (<1%) were excluded from analysis. A total of 10.8% were missing either FGM/C status or a completed scale (*n* = 69); however, missingness in the scale was not associated with FGM/C status, nor was it associated with the mental or physical health outcome variables, indicating the potential that excluding incomplete cases is less likely to introduce bias. It is possible that some women did not wish to disclose their FGM/C status due to the illegality of the practice in the U.S.

Perceived discrimination was measured using the Everyday Discrimination Scale short version [49, 50]. Response options for each of the five scale items, referring to possible experiences of discrimination, ranged from 1 “never” to 6 “almost everyday.” Participants then selected the perceived reason(s) for these experiences from a predetermined set of options. Examples of possible experiences included the perception of being treated with less courtesy or respect than other people, people acting as if they are afraid of them, and feeling threatened or harassed. Participants missing data for one or more scale items (<3%) were excluded from analysis. This scale displayed good internal reliability with a Cronbach’s alpha of 0.94. A total summed scale score and mean item score were calculated. The reliability and convergent validity of this scale was previously determined to be strong when used in a population of resettled adolescent Somali refugees in the U.S. [51].

### Analysis

Descriptive statistics included reports of frequencies or means and standard deviations for variables of interest. Bivariate logistic regression analyses explored potential associations between FGM/C and the three key categories of variables, (1) social experiences: perceived social support and everyday discrimination, (2) identity and heritage: time lived in the US and preferences for living and socializing within ethnic enclaves, and (3) health outcomes: mental distress and having experienced a sexual, obstetric or gynecological health concern in the last two years (for timepoint 2 data), or ever experienced (for baseline data). Multivariable logistic models explored whether either of the two health outcomes were associated with FGM/C status when accounting for these other factors. Age and educational attainment were controlled for in the models based on the theoretical assumption that these variables would potentially be related both to the health outcomes and to other independent variable in the model (i.e. a relationship between educational attainment and age). Potential collinearity was assessed by checking variance inflation factors and cross tabulations to explore relationships between the independent variables. No significant collinearity was present. Statistical analyses were performed in STATA 16 [52].

## Results

Participants (*n* = 638) included those who reported FGM/C status at baseline and participated in a wave 2 survey. In both the waves 1 and 2 surveys, approximately 80% of participants reported FGM/C (Table 1). There were overarching demographic differences between “FGM/C” and ‘No FGM/C’ women. ‘No FGM/C’ Somali women were younger (M = 22.84, SD = 8.86) than ‘FGM/C’ women (M = 33.32, SD = 13.96), *t[830]* = –8.99, *p* = 0.00, Cohen’s d = –0.80. While nearly half of ‘FGM/C’ women had either attended only primary school or never attended school, over 80% of ‘No FGM/C’ women had attended high school or greater educational attainment. Approximately one quarter of ‘No FGM/C’ women attended college compared to less than 15% of ‘FGM/C’ women.

**Table 1:** Characteristics of Somali women participating in baseline (*n* = 847) and follow up (*n* = 638) surveys.

	Baseline *N* = 847 n (%)	Follow up *N* = 638 n (%)		
FGM/C				
Yes	686 (81.0)		514 (80.6)	
No	161 (19.0)		124 (19.4)	
	No FGM/C (N %), Mean (SD)	FGM/C (N%), Mean (SD)	No FGM/C (N %), Mean (SD)	FGM/C (N%), Mean (SD)
Age (years)	22.84 (8.86)	33.32 (13.96)	24.54 (8.79)	35.15 (14.46)
Ethnicity				
Northern Somali	40 (24.84)	112 (16.33)	27 (21.77)	68 (13.23)
Southern Somali	39 (24.22)	374 (54.52)	29 (23.39)	263 (51.17)
Somali Bantu	55 (34.16)	158 (25.03)	48 (38.71)	146 (28.40)
Other	23 (14.29)	29 (4.23)	17 (13.71)	26 (5.06)
Missing	4 (2.48)	13 (1.90)	3 (2.42)	11 (2.14)
Educational attainment				
Never attended	27 (16.77)	181 (26.38)	13 (10.48)	148 (28.79)
Primary	14 (8.70)	122 (17.78)	9 (7.26)	86 (16.73)
Some high school	47 (29.19)	146 (21.28)	38 (30.65)	99 (19.26)
High school diploma	26 (16.15)	130 (18.95)	23 (18.55)	87 (16.93)
Some college	29 (18.01)	60 (8.75)	27 (21.77)	45 (8.75)
College/advanced degree	13 (8.07)	36 (5.25)	6 (4.84)	30 (5.84)
Missing	5 (3.11)	11 (1.60)	8 (6.45)	19 (3.70)

### FGM/C, discrimination, and perceived social support

Women reported low average item scores on the Everyday Discrimination Scale (Table 2), both those with FGM/C (M = 1.54, SD = 0.92) and those without (M = 1.74, SD = 1.05). ‘No FGM/C’ women reported more frequently experiencing discrimination (defined as experiencing a particular discrimination type a few times a month, at least once a week, or almost every day) across the five types of discrimination as compared to ‘FGM/C’ women: specifically, 23.2% of ‘No FGM/C’ women and 7.6% of ‘FGM/C’ women reported experiencing two or more types of discrimination on a frequent basis.

**Table 2:** Everyday discrimination among Somali Women with FGM/C (*n* = 498) and without FGM/C (*n* = 121) and Perceived Social Support among Somali Women with FGM/C (*n* = 470) and without FGM/C (*n* = 99).

Everyday Discrimination						
	No FGM/C (*n* = 121) M(SD)			FGM/C (*n* = 498) M(SD)		
Average discrimination item score (1–6, Never—Almost Every day)	1.74 (1.05)			1.54 (0.92)		
Discrimination total scale score (5–30)	8.68 (5.26)			7.70 (4.57)		
Types of discrimination						
	NeverN (%)	Infrequent^a^ N (%)	Frequent^b^ N (%)	NeverN (%)	Infrequent N (%)	Frequent N (%)
1. I was treated with less courtesy than others	87 (71.9)	26 (21.5)	8 (6.6)	346 (69.5)	129 (25.9)	23 (4.62)
2. I received poorer service than others	76 (62.8)	22 (18.2)	23 (19.0)	355 (71.3)	105 (21.1)	38 (7.6)
3. People acted as if they thought I am not smart	81 (66.9)	29 (24.0)	11 (9.1)	353 (70.9)	110 (22.1)	35 (7.0)
4. People acted as if they were afraid of me	79 (65.3)	23 (19.0)	19 (15.7)	355 (71.3)	108 (21.7)	35 (7.0)
5. I was threatened or harassed	84 (69.4)	17 (14.1)	20 (16.5)	385 (77.3)	92 (18.5)	21 (4.2)
Total number of discrimination types experienced FREQUENTLY (a few times per month or more) (out of 5 possible areas of discrimination)	No FGM/C N(%)			FGM/C N(%)		
0	89 (73.6)			433 (87.0)		
1	4 (3.3)			27 (5.4)		
2	14 (11.6)			11 (2.2)		
3	10 (8.3)			14 (2.8)		
4	1 (0.8)			4 (0.8)		
5	3 (2.5)			9 (1.8)		
What do you think is the main reason for these experiences? (select all that apply)						
	No FGM/C N(%)			FGM/C N(%)		
Your clan or ethnic group/affiliation	27 (22.3)			37 (7.4)		
Your gender	21 (17.4)			34 (6.8)		
Your ancestry or national origins	20 (16.5)			43 (8.6)		
Your race	17 (14.1)			36 (7.2)		
Your religion	16 (13.2)			45 (9.0)		
Your age	9 (7.4)			11 (2.2)		
Your education or income level	4 (3.3)			10 (2.0)		
Your height	2 (1.7)			6 (1.2)		
Your weight	1 (0.8)			5 (1.0)		
Your appearance	0 (0.0)			8 (1.6)		
Your sexual orientation	0 (0.0)			5 (1.0)		
Number of different reasons they believed they experienced discrimination						
0 (no reason selected)	89 (73.6)			428 (85.9)		
1	0 (0.0)			8 (1.6)		
2–4	25 (20.7)			40 (8.0)		
5 +	7 (5.8)			22 (4.4)		
Perceived Social Support						
	No FGM/C M(SD) (*n* = 99)			FGM/C M(SD) (*n* = 470)		
Average MSPSS item score (range: 1 “strongly disagree” – 5 “strong agree”)	3.92 (1.03)			4.09 (0.86)		
Significant Other Subscale average item score	3.97 (1.12)			4.17 (0.95)		
Family subscale average item score	4.02 (1.09)			4.19 (0.93)		
Friends subscale average item score	3.78 (1.06)			3.92 (0.94)		

^a^”Infrequent” defined as a few times a year or less than once a year.

^b^”Frequent” defined as a few times a month, at least once a week, or almost everyday.

Among all women who responded to the wave 2 survey, religion was the most cited perceived cause of discrimination (*n* = 72, 11.3%). Among ‘No FGM/C’ women, the top five perceived causes of discrimination were clan/ethnicity (22.3%), gender (17.4%), ancestry/nationality (16.5%), race (14.1%), and religion (13.2%). Over one quarter of all ‘No FGM/C’ women and over 10% of ‘FGM/C’ women thought that their discrimination experiences were due to two or more causes.

Perceived social support was high among both ‘No FGM/C’ (M = 3.92, SD = 1.03) and ‘FGM/C’ (M = 4.09, SD = 0.86) women (Table 2). The highest item average scores for both groups were within the Family Subscale, with lower scores in the Friends Subscale.

Bivariate associations between FGM/C status and these social experiences (Table 3) revealed that women with FGM/C were 18% less likely to report everyday discrimination experiences (OR = 0.82, CI: 0.67-0.99) compared to women who did not undergo FGM/C. No significant difference was found in perceived social support based on FGM/C status.

**Table 3:** Bivariate associations between FGM/C status and everyday discrimination (*n* = 619) and social support (*n* = 569) among Somali women.

	Odds ratio (95% CI)
Average discrimination item score	0.82 (0.67–0.99)^*^
Average MSPSS item score	1.22 (0.97–1.55)

*P value < 0.05

### Health outcomes

Among the 576 women who completed the RHS-13 in its entirety, 128 women (20.0%) screened positive on the RHS-13 for clinically significant symptoms of distress (Table 4). While proportionally more “No FGM/C” women (27.0%) than ‘FGM/C’ women (18.9%) screened positive, this relationship was not statistically significant in bivariate analysis. One hundred and six women (16.6%) reported having experienced one or more negative health events of a sexual, obstetric, or gynecological nature over the last two years. The relationship between having experienced one or more of these health events and FGM/C status was not significant (OR = 0.79, CI:0.48–1.31).

**Table 4:** Bivariate associations between FGM/C status and mental (*n* = 576) and physical (*n* = 638) health among Somali women with FGM/C.

	No FGM/C N(%)	FGM/C N(%)	Odds ratio (95% CI)
RHS-13 Refugee Health Screen			
Negative	81 (73.0)	377 (81.1)	0.63 (0.39–1.02)
Positive	30 (27.0)	88 (18.9)	
Reported experiencing 1+ sexual, obstetric or gynecological health problems in last two years			
No	100 (80.7)	432 (84.1)	0.79 (0.48–1.31)
Yes	24 (19.4)	82 (16.0)	

*P value < 0.05

For the outcome of a positive screen for clinically significant psychological distress, multivariable logistic regression controlling for age, ethnicity, and educational attainment, and including the item average scores for both perceived discrimination and perceived social support, revealed that FGM/C status was not significantly associated with distress (Table 5). Those reporting higher everyday discrimination were five times as likely to screen positive for distress (OR = 5.11, CI: 3.63–7.20). Perceived social support appears protective against psychological distress in this model, with those reporting higher support being 43% less likely to screen positive for distress (OR = 0.57, CI: 0.41–0.82).

**Table 5:** Multivariate regression models of FGM/C status, demographic and social experience variables among Somali women onto outcomes of psychological distress (*n* = 495) and reported sexual/gynecological/obstetric health (*n* = 529).

Model 1. Outcome = Positive RHS-13 Screen	OR (95% CI)
Age (years)	1.04 (1.01–1.06)^*^
Ethnicity	
Northern Somali	Ref
Southern Somali	0.89 (0.34–2.30)
Somali Bantu	1.51 (0.56–4.12)
Other	1.41 (0.21–9.32)
Educational attainment	
Never attended	Ref
Primary	0.27 (0.10–0.74)^*^
Some high school	0.45 (0.17–1.20)
High school diploma	0.56 (0.21–1.56)
Some college	0.30 (0.08–1.17)
College/advanced degree	1.28 (0.34–4.86)
FGM/C	
No	Ref
Yes	0.95 (0.38–2.37)
Everyday Discrimination average item score	5.11 (3.63–7.20)^*^
Perceived Social Support average item score	0.57 (0.41–0.82)^*^
**Model 2. Outcome = Reported sexual, gynecological or obstetric health outcome in past two years**	**OR (95% CI)**
Age (years)	1.01 (0.99–1.03)
Ethnicity	
Northern Somali	Ref
Southern Somali	0.54 (0.25–1.17)
Somali Bantu	0.87 (0.39–1.94)
Other	0.75 (0.18–3.09)
Educational attainment	
Never attended	Ref
Primary	1.95 (0.86–4.42)
Some high school	1.09 (0.45–2.63)
High school diploma	1.35 (0.56–3.30)
Some college	1.52 (0.50–4.56)
College/advanced degree	2.94 (0.92–9.33)
FGM/C	
No	Ref
Yes	1.05 (0.52–2.10)
Everyday Discrimination average item score	2.68 (2.10–3.43)^*^
Perceived Social Support average item score	0.72 (0.54–0.97)^*^

*P value < 0.05

For the outcome of having experienced one or more sexual, obstetric, or gynecological health events in the past two years, controlling for and including these same variables, women reporting greater everyday discrimination were more than two and half times as likely to experience a sexual, obstetric, or gynecological health event over the last two years (OR = 2.68, CI: 2.10–3.43). Women reporting greater perceived social support were 28% less likely to report this health outcome (OR = 0.72, CI: 0.54–0.97).

## Discussion

Our findings are among the first to quantitatively elucidate the lived experience of women from FGM/C practicing communities among a culturally distinct yet racialized minority in the United States. Somali women living in Arizona who did not undergo FGM/C were more likely to be younger, more educated, and more likely to report discriminatory experiences compared to FGM/C-affected Somali women. Social support was high in this population, regardless of FGM/C status. Finally, while FGM/C is associated with physical and psychological health morbidities in the literature, this study finds that FGM/C status was not significantly associated with perceived psychological distress or experiencing a sexual, gynecologic, or obstetric health morbidity in the past two years. In fact, discrimination was the shared predictor of poor physical and mental health, with social support playing a key protective role against distress.

The 2017 Executive Order by then-U.S. President Donald Trump restricting travel (known as the “Travel Ban” or “Muslim Ban”), targeted Somalia (amongst a host of other predominantly Muslim countries), and has been argued to constitute an Islamophobic policy, despite being applied under the pretense of homeland security. Under the backdrop of the Black freedom struggle, such a volatile context has led to a surge in anti-Somali rhetoric [53, 54], and hate crimes [55]. As Jesse Mills states, “the legacy of race, gender, and class oppression in the U.S. has transported many Somali refugees from one epic struggle to another” [56]. Such political tactics have detrimental effects on women’s well-being. For example, research indicates that the so-called Muslim ban was associated with a significant increase in preterm birth for women originating from targeted countries, living in the U.S. [57] and a significant decrease in healthcare services utilization in the U.S., by targeted communities (i.e., missed primary care appointments and increased emergency department visits) [58]. These outcomes were particularly likely among women facing interlocking systems of oppression related to their gender, race, migration status, and FGM/C status. Being a racialized “other” has a significant impact on one’s sense of belonging. Divisive politics and fear-mongering propaganda around alleged Somali terrorism have ostracized an already marginalized community and one may posit that they further compromised the fragile trust between the Somali community and public health institutions [59, 34].

The present globalized context of social polarization and hostility around Islam has placed several marginalized migrant communities under much duress [60–63] and the precipitous rise in Islamophobic attacks since 9/11 are a public health concern [64]. This polarization and hostility has cast the Muslim body as both a perplexity and a threat, which has served to legitimize anti-migrant and Islamophobic policies and rhetoric across the U.S. (along with a host of other countries) [65, 66], in line with structural racism [67].

Perhaps in part due to discrimination based on their migrant status and religion, Somali individuals (especially in FGM/C-affected communities) have reported feeling “not normal” and “different” in their Western host country [68] a notion perpetuated by discriminatory public discourse depicting the “barbaric” nature of FGM/C and related cultures [69]. A critical discourse analysis of Somali men and women found that Somalis described experiencing discrimination regarding their Muslim identities and that these experiences had perceived impacts on their use of health services [35]. Moreover, Somalis have been noted to be at increased risk of being targets of discrimination compared to other migrant groups due to the *‘triple jeopardy’* intersection of being Black, refugees and predominantly Muslim [70]. Whereas race, ethnicity, and religion (in the case of wearing hijab) may be easily recognizable in the public space, FGM/C is not. It is possible that these social discourses linking FGM/C to Islam in general, and Somalis in particular, provides additional fuel for discrimination, regardless of whether the woman in question is affected by FGM/C (a fact that cannot be known by the casual observer).

While the Somali community is largely comprised of resettled refugees, our sample was more diverse, representing the broader diaspora of Somali migrants, encompassing various migratory pathways beyond refugee resettlement. The finding that young, educated, ‘No FGM/C’ Somali women reported experiencing higher rates of discrimination should not be lost on us. Evidence shows that FGM/C practices are largely abandoned after moving to the Global North [71, 72]. While we must celebrate the fact that young Somali women are remaining unaffected by FGM/C and are afforded educational opportunities in the U.S., we must also explore how their lived experiences as young, educated, FGM/C-unaffected Somali women may increase their risk of harmful discriminatory interactions. The finding from the final model that having received a primary school education, as opposed to no schooling, was negatively associated with psychological distress when controlling for age may indicate that there is indeed some overall benefit to educational attainment when it comes to psychological distress among women in this population.

Our data were collected during the Executive Order, and hence it follows that our participants identified religion, origins, and ethnicity as the main sources of discrimination they endured. These considerations may explain why discrimination, and not FGM/C status, were the driving predictor of poor health in this analysis. This finding is quite compelling considering the widespread notion that FGM/C irrefutably and systematically leads to trauma and mental illness, such as anxiety and depression. However, it is important to consider the ways in which distress related to vivid recollection of the original FGM/C experience can be triggered when women traverse social systems such as health care [34]. For example, women with FGM/C in Canada have reported such recollection elicited by vulvar/vaginal examinations, in which their doctors did not know how to care for their resultant emotional responses [69]. Similarly, West African women with FGM/C reported negative and insensitive reactions from medical professionals regarding their FGM/C status that created feelings of shame [7]. The performance of surgical vulvar reconstructive procedures such as defibulation (for Type III FGM/C) and clitoral reconstruction (for any form of clitoral excision performed in Types I, II, or III FGM/C), while potentially ameliorating some obstetric, gynecologic, or sexual morbidity in women, has been demonstrated to also trigger post-traumatic stress at times [73, 74]. While women’s distress may seem directly related to FGM/C, what must also be considered is the health care system’s under-preparedness to care for women with FGM/C and conditions prominent in Black communities—a notion reported by women with FGM/C themselves [69] and by their service providers [75, 76]. This is in line with our results, which reveal that while FGM/C alone does not independently predict perceived psychological distress, discrimination does. While discrimination and the healthcare experience may play a large role in current self-reported psychological distress, prior analyses have indeed shown that when looking specifically among only those Somali women in the U.S. with FGM/C, recalling the FGM/C event as being traumatic is also associated with distress [34].

Our transnational colonial legacy established a culture of imperialism, exploitation, exoticization, and subjugation that cannot be extricated from the endurance of present-day racism and discrimination against communities of color, including African migrant populations [15]. Postcolonial discourse must recognize the intersection of gender and race within the global as well as U.S. context, which has a long history of oppression, objectification, hypersexualization, medical experimentation, and exploitation of Black women’s bodies [77]. In a notable example, Sarah Baartman was paraded throughout Europe during the early 19th century as the *“Hottentot Venus”* and displayed in cages where people paid extra to poke and prod her genitalia and buttocks, promulgating ideas of sexual primitivism inherent in Black women [78]. In the U.S., Black bodies were deemed impervious to pain, as J. Marion Sims performed countless gynecologic procedures on enslaved Black women without their consent or anesthesia—a series of experiments from which modern gynecologic surgery was born [79]. The term *‘Mississippi Appendectomy’* was coined by Fanny Lou Hamer to codify the rampant coerced sterilization procedures that were performed throughout the Eugenics Movement from the 1920s–1980s on over 8000 Women of Color throughout the U.S. South [80]; involuntary or inadequately consented sterilization continues to the present day among migrant women across U.S. detention facilities [81]. It is against this backdrop that FGM/C-affected women must navigate health care encounters, often involving an “*othering*”medical gaze, and negative care experiences amidst threats to their own personhood. It is no wonder that the literature abounds with evidence of profound fear of the health care system, distrust, delay in health care-seeking, and resultant adverse health outcomes [82]. Consequently, any critical discourse on the intersectionality of FGM/C must be considered within the enduring historical colonial and postcolonial legacies of Western cultural hegemonic domination [15].

A growing body of evidence also demonstrates the effects of SDoH on communities of color that possess fewer material resources and access to power, putting them at greater risk for mental disorders and poorer mental health [83]. Indeed, a context marred by social inequities has a profound bearing on health. Experiences of social exclusion, perception of powerlessness and stress influence neuroendocrine, epigenetic and neuroimmune responses, which in turn have an impact on physical and mental health [84]. While FGM/C can lead to psychological distress, such an outcome is not a foregone conclusion [85, 86]. For instance, compared to Somali women who have undergone FGM/C, those who have been exposed to violent crimes such as homicide, sexual assault, and kidnapping, are four times more likely to experience depression or trauma [30]. This research illustrates the problem of cumulative trauma, the relevance of delving into pre- and post-migration events, and considering women’s life stories beyond the experience of FGM/C.

In the face of this complex marginalization in the U.S., which includes social, political, historical, economical, migratory, legal, and policy-based considerations, social support may act as a bulwark against distress. The conceptualization of cultural and ethnic identity is malleable and shaped in part by the sociopolitical landscape and mainstream media depiction of migrant groups. Studies conducted in the U.S. indicate that the salience of racial identity among Somali refugees increases drastically following resettlement in the U.S. [87, 88], where racial tensions are more conspicuous than in Somalia, which shows more ethnic homogeneity. Additionally, studies conducted on the 2017 Travel Ban also reveal profound feelings of disempowerment and fear of racist attacks in the Somali community [89]. North American public discourses (i.e., anti-FGM discourses), though well-intentioned, often further ‘other’ and even defame FGM/C-affected communities and cultures under the banner of human-rights advocacy, increasing the work such communities must engage in to prepare for encounters with health systems, which they fear will perpetuate the same discrimination [69, 90–92]. It is now well-established that SDoH are a significant driver of population health inequities [83, 93] and that racism is a public health crisis [94, 95]. Our results suggest that social ties and feelings of affiliation within a community can serve as a counterpoint to perceived racism and discrimination and correlate positively with psychological wellbeing. Further analyses into the specific role of perceived social support and an unpacking of its processes in the health of this population is warranted, considering that Somali American communities are both diverse and dynamic and social support systems may be impacted both by acculturation and in-country relocation after resettlement.

While “physical appearance” was offered as a possible reason for discrimination, FGM/C was not itself included. This limits the inferences we can make regarding the connection between discrimination and FGM/C-status. Furthermore, snowball sampling may have potentially impacted the representativeness of this sample, and therefore the generalizability of these results cannot be assured. Future studies may benefit from additional statistical methods better able to tease out the precise relationships between the variables studied. While the instruments regarding discrimination and social support were not adapted specifically for use in this population, the instruments along with all items in the survey were vetted through community input and found to be appropriate.

## Conclusion

Discrimination underscores a *‘quadruple jeopardy’* of intersectional relationships encompassing gender, race, religion, and migration that bring adverse health consequences to Somali migrant communities, which social support may help mitigate. By shedding light on the intersectionalities and sociopolitical factors at play, the results of this study are an invitation to reframe our understanding of FGM/C’s impact on women’s health and well-being through the lens of postcolonial African diasporic feminist discourse. FGM/C undoubtedly impacts physical health morbidity in certain contexts, depending on the type and severity of cutting, the skill of the practitioner, the use or disuse of adequately sterilized instruments, and so on. Indeed, health risks apply to all forms of genital cutting, including forms that are widely accepted in Western culture, such as so-called “cosmetic” genital surgeries that are not treated as mutilations, despite overlapping anatomical outcomes [96]. Concerning mental health, outcomes vary considerably across types of genital cutting depending on the immediate circumstances of the procedure, the wider social context, and the experiences, beliefs, psychological attributes, and attitudes (i.e., toward the cutting or its bodily or symbolic significance) of the affected individual [97]. What is necessary to keep in mind is that devastating physical and mental health outcomes must not be simply assumed to follow from FGM/C, as this may not be the primary driver for poor health in many cases.

Considering the potentially changing demographics of Somali-American women over time and across the life course, and the likelihood that social factors such as discrimination and support may play a larger role in health than FGM/C, we must perhaps question our own motives in public health in continuing to focus on FGM/C in isolation from its social and political context. Indeed, communities where FGM/C exists may provide spaces of strong social support and belonging that are of benefit to women regardless of their FGM/C status. Future research is needed to further deconstruct the intersectional relationships that remain at the core of persistent and systemic health inequities among communities of color, including migrant populations. In so doing, we may better understand how women from FGM/C-practicing communities, whether affected by FGM/C or not, navigate their health within the complexity of their emerging Western identities.

## Data Availability

Due to the sensitive manner through which longstanding community trust was nurtured and sustained, data was collected while partnering with a vulnerable and marginalized community amidst a sociopolitical context which historically harmed this community. Consequently, data may only be made available upon request to the Corresponding Author in a collaborative venture due to concerns for the potential of unintended harms exacted by how the data may be used, interpreted, and disseminated.

## Footnotes

1 Indeed, even the term “FGM/C”—which we reluctantly adopt as a compromise between “female genital mutilation” and “female genital cutting”—reflects dominant Western attitudes toward the set of practices in question. Numerous African languages use the same general terminology for the genital cutting of women and men [11] but have their own vernacular and cutting classifications, which account for variations in the way different ethnic groups perform FGM/C [12]. Considering the hegemonic global forces undergirding the perpetuation of “FGM/C” as a concept, the terminology becomes political and raises questions about who has the power to name phenomena or cultural practices [13]. While the terminology continues to be a source of controversy and has evolved over time and throughout different UN agencies [14], the term FGM/C is used in the present paper.

2 The term FGM/C-affected communities is a broad umbrella term which references communities for which FGM/C is a notable practice or ‘cultural marker’ of that community, even if some individuals within that community may not have experienced the practice.
